# Accuracy of Triage Nurses in Predicting Patient Admissions: Retrospective, Large-sample Evidence from a Community Emergency Department

**DOI:** 10.5811/westjem.39947

**Published:** 2025-09-01

**Authors:** Calvin Armstrong, David Kanter-Eivin, Michaela Dowling, Grant Sweeny, Asil El Galad, Anil Esleben, Nanda Krishna Duggirala, Corrine Mitges, Shauna Speck, Stephenson Strobel

**Affiliations:** * McMaster University, Michael G. DeGroote School of Medicine, Hamilton, Ontario, Canada; † Niagara Health, Ontario, Canada; ‡ McMaster University, Division of Emergency Medicine, Department of Family Medicine, Faculty of Health Sciences, Hamilton, Ontario, Canada

## Abstract

**Introduction:**

Emergency department (ED) flow could be improved with quicker disposition decisions. One possible way to expedite decisions is for triage nurses to make predictions about whether patients require admission to hospital. The information contained in these predictions could be useful for disposition planning and for physician decision-making. Previous studies made use of prospective designs that introduced Hawthorne effects and have demonstrated mixed evidence on whether triage nurse predictions are accurate. We examined the accuracy of triage nurse predictions for patient admission in an ED in southeastern Ontario.

**Methods:**

We examined a retrospective sample of 134,891 visits to an ED in Ontario from March 2019 – July 2024. Triage nurses made predictions about admission to hospital for these visits, from which we estimated measures of specificity, sensitivity, positive predictive value, negative predictive value, accuracy, and F_1_ scores.

**Results:**

Of 134,891 visits, 13.7% resulted in hospital admission. We found the accuracy of the nurses in predicting admission to be 85.8% (95% confidence interval [CI] 85.7 – 86.1), while overall sensitivity was 36.6% (95% CI 35.9 – 37.3) and specificity was 93.7% (95% CI 93.5 – 93.8). The positive predictive value of admission was 47.9% (95% CI 47.1 – 48.7), and the negative predictive value of admission was 90.3% (95% CI 90.1 – 90.5). F_1_ scores were 0.415. These results were relatively stable over time, although there was notable variation in prediction ability between nurses. We also report that some presenting conditions lead to relatively higher prediction accuracy than others and that as overall case severity increases, sensitivity increases and specificity decreases.

**Conclusion:**

These results suggest that although nursing staff predictions are insufficient to streamline disposition decisions completely, they could be useful in expediting certain decisions related to hospital admission and resource requirement, thereby improving flow in EDs.

## INTRODUCTION

Emergency departments (ED) face prolonged patient wait times and crowding due to limited resources. Despite goals of reducing wait times, patients in the province of Ontario, Canada, waited an average of 30 minutes longer in 2022/2023 than in 2013/2014—up to an average of 118 minutes—to see an emergency physician. 1 In the United States, only a minority of hospitals consistently achieve recommended wait times for all patients, 2 although this has improved over time. 3 Extended ED wait times lead to patient dissatisfaction, 4 patients leaving without being seen by a physician, 1 poorer outcomes, and higher resource use per admission to hospital. 5 Crowding has also been linked to increased patient mortality. 6–8

A key challenge to improving ED wait times and crowding is flow: how can clinicians make quick yet accurate decisions about disposition of patients to hospital or discharge? Quicker disposition means freeing up resources such as beds and monitoring staff for other ED patients. 9 However, disposition decisions require the emergency physician’s time, which is one of the most scarce resources in an ED. One possible solution is to leverage the skill of triage nurses in identifying patients who require more involved care. 10 Using triage nurses to predict whether patients require admission could streamline resources toward high-risk patients, alert specialist physicians to patients who might require hospital care, and provide emergency physicians quick information to make quicker disposition decisions.

The success of this solution depends on ED nurses’ ability to make accurate predictions about patient disposition. Previous literature highlights notable variability in the accuracy of nursing predictions. Several studies report an accuracy rate of 70% or higher for predicting patient disposition or outcome, with nearly 90% accuracy for predicting patient discharge. 11–13 Other research contradicts these findings, with one study demonstrating inappropriate patient triaging in >40% of patient presentations. 14 There is also variability regarding the factors that influence nurses’ prediction accuracy. Some studies demonstrate a positive correlation between nurse experience and predictive capability, 15 while others do not. 11,16 Certain patient characteristics, including age and severity of presentation, have been correlated with high predictive accuracy, although other literature has failed to replicate these findings. 12,13

Most existing studies on nurse prediction rely on prospective designs, which limit sample size and make predictions prone to Hawthorne effects where subjects of studies change their behavior because they are being observed. 17 In contrast, we examined an ED operations change that required triage nurses to predict whether a patient required admission to facilitate earlier involvement of allied health. Our study makes two key contributions to the literature: 1) unlike prospective research, our results better reflect real-world conditions, providing evidence on how triage nurses predict “in the wild” when they do not think they are being studied; and 2) by leveraging a large set of high-quality administrative data, we were able to explore the nuances of triage nurse predictions. This enabled us to examine heterogeneity in prediction accuracy by patient type and other important characteristics. Our study adds to the literature on nurses’ predictive capabilities and to a smaller body of literature examining the heterogeneity in prediction by nurse and patient type.

**Population Health Research Capsule**

What do we already know about this issue?

*Previous literature has reported conflicting evidence about the ability of emergency department (ED) triage nurses to predict patient admission requirement*.

What was the research question?

*Can triage nurses accurately predict patient hospital admissions in a real-world, community ED?*

What was the major finding of the study?

*Nurse prediction accuracy was 85.8% (95% CI 85.7 – 86.1), with sensitivity 36.6%, specificity 93.7%, and F_1_ score 0.415*.

How does this improve population health?

*Triage nurse predictions may help fast-track care for certain patients, reducing ED delays and optimizing hospital resource use*.

## MATERIALS AND METHODS

### Study Design

This was a retrospective cohort study that used administrative health data collected during March 2019 – July 2024.

### Setting

We conducted the study using data from a community ED within the Niagara Health system in southeastern Ontario. This site sees 80 – 100 patients per day and 30,000 – 40,000 visits per year. The ED has approximately 20 – 30 regular nurses who are able to triage.

As part of a quality improvement (QI) initiative, triage nurses were asked to indicate within the electronic health record (EHR) system if they believed a patient would require hospital admission. Predictions began as a QI initiative to reduce potential admission time. Triage nurses flagged patients whom they thought would be admitted so that allied healthcare like occupational therapy and discharge planning would quickly see patients who likely needed their services. Triage nurses received no specific training. For each triaged patient an additional question, “predicted admission y/n,” was added to the triaging screen after the nurse recorded the patient’s past medical history. Prediction could not be routinely bypassed except for rapidly evolving emergencies or when the EHR was down for maintenance. There were also exceptions for agency nurses who had not been hired full-time. For these situations nurses could triage by paper and a prediction was not entered into the EHR.

This administrative data allowed us to measure admission outcomes and define surrogates for admissions to test how accurate nurses are at predicting. Specifically, in our primary outcome, we included the following as an admission:

1. Any admission to the hospital at the time of the index ED visit
2. Transfers to alternate hospitals
3. Deaths in the ED.

We also considered patients who returned to the ED for any reason within 30 days and subsequently required admission (or met one of the above criteria) as “admissions” for the purpose of evaluating prediction accuracy. Using this surrogate, we attempted to measure inappropriate discharges (i.e., patients who should have been admitted but were not) by the physician at the index visit. Our rationale for this broad, 30 - day window was that even if the return visit was for a seemingly unrelated issue, the need for admission indicated a potential clinical necessity that might not have been fully recognized at the initial presentation.

### Outcome Measures

Our main measure of interest was the accuracy of a nurse predicting admission to hospital. We measured this by estimating sensitivity (1) and specificity (2) of admissions predictions. 18 These are defined as


(1) $$Sensitivity\;(TPR)=\frac{TP}{TP+FN}$$

and


(2) $$Specificity\;(TNR)=\frac{TN}{TN+FP}$$

We also provided estimates for (3) positive predictive value and (4) negative predictive value defined as


(3) $$Positive\;Predictive\;Value\;(PPV)=\frac{TP}{TP+FP}$$

and


(4) $$Negative\;Predictive\;Value\;(NPV)=\frac{TN}{TP+FN}$$

We treated the emergency physician’s decision to admit as the reference standard, supplemented by the admission surrogates noted above. The components of these measures were as follows:

- **True positives (TP):** Patients predicted to need admission who are admitted, transferred, die in the ED, or return to the ED within 30 days (for any reason) and met one of these criteria.
- **False negatives (FN):** Patients predicted not to need admission but who were admitted, transferred, died in the ED, or returned to the ED within 30 days (for any reason) and met one of these criteria.
- **True negatives (TN):** Patients predicted not to need admission and who did not meet any of the above criteria at the index visit or within 30 days.
- **False positives (FP):** Patients predicted to need admission but did not meet any of the above criteria and did not return within 30 days requiring admission.

We also evaluate overall accuracy, defined as


(5) $$Accuracy\;(ACC)=\frac{TP+TN}{TP+TN+FP+FN}$$

Finally, because substantially more patients are discharged than admitted, we also calculated the F_1_ score, which balances sensitivity (recall) and positive predictive value (precision). This is commonly used in machine-learning and is useful in settings with imbalanced outcomes, 19 such as ED visits where admissions are less common. The F_1_ score is determined by the following calculation:


(6) $$F_{1}=\frac{TP}{TP+\frac{1}{2}(FP+FN)}$$

F_1_ scores below 0.5 are considered poor, and scores between 0.5 and 0.8 are considered average.

### Additional Analyses

We also estimated a prediction compliance rate as the number of predictions that are recorded over the total number of patients. We provided several extensions of our headline measurements of compliance, namely specificity, sensitivity, positive predictive value (PPV), negative predictive value (NPV), accuracy, and F_1_ score. First, we examined how stable these outcomes have been over the period of observation, to see whether predictions vary with familiarity. Second, we examined the inter-nurse variation in predictions to check whether some nurses predict better than others. Finally, we examined whether prediction outcomes varied by a patient’s assigned triage acuity score and patient complaint. The former variable, the Canadian Triage and Acuity Scale (CTAS), is a computer-calculated measurement of the patient’s requirement for acute resources and corresponds to sickness of the patient. 20 The CTAS categories correspond to a scale of 1–5, specifically resuscitation (1), emergent (2), urgent (3), less urgent (4), and non-urgent visits (5).

Finally, To examine how our definition of “admission” (which includes 30-day readmissions for any reason) impacts our results, we also perform a sensitivity analysis that alters the outcome so that it only 1) includes seven-day readmissions, 2) one day readmissions and 3) excludes these return admissions entirely (i.e., only includes the index visit).

### Inclusion and Exclusion Criteria

We made two data restrictions when examining prediction heterogeneity to avoid small sample sizes. For inter-nurse prediction, we only included nurses who registered ≥ 50 predictions over the study period. Our examination of nurse heterogeneity was also restricted to the period of January 2020 – July 2024, as we did not have information on which nurses made predictions prior to this. For examination of presenting complaints, we only included predictions for complaints that appeared at least 100 times throughout the study period.

### Data Analysis

Analysis was performed with Stata 18 (StataCorp, LLC, College Station, TX). For our overall parameters of sensitivity, specificity, positive predictive value and negative predictive value, we provide a 95% confidence interval (CI) that is based on a two-sided test.

### Ethics Approval

Ethics approval was obtained through the Hamilton Integrated Research Ethics Board under project number 17330.

## RESULTS

**Figure 1:**
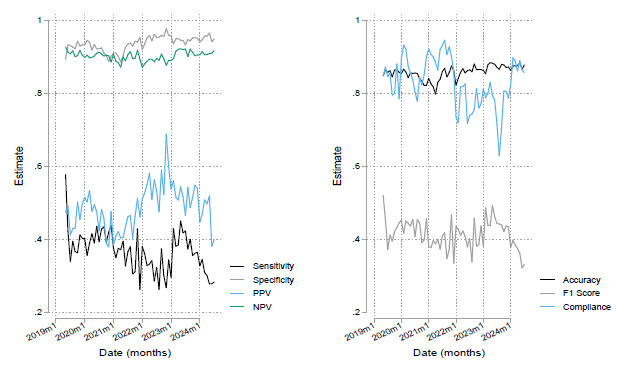
Time series of outcomes of interest over the period of observation. *PPV*, positive predictive value; *NPV*, negative predictive value.

During the study period of March 2019 – July 2024, 162,392 visits occurred at the ED. Triage nurses provided disposition predictions for 134,891 visits for an overall compliance rate of 83%. Of these visits, 16,022 resulted in admission to the hospital. Nurses correctly predicted 6,764 admissions (TP) but missed 11,700 admissions (FN), resulting in an overall sensitivity of 36.6% (95% CI 35.9 – 37.3). Additionally, triage nurses accurately predicted that 109,067 visits would not result in an admission (TN), while 7,360 visits that they predicted as admissions did not result in hospitalization (FP), yielding a specificity of 93.7% (95% CI 93.5 – 93.8). These findings correspond to positive and negative predictive values of 47.9% (95% CI 47.1 – 48.7) and 90.3% (95% CI: 90.1, 90.5), respectively. The nurses’ overall accuracy during the period of observation was 85.8% (95% CI 85.7 – 86.1). The F_1_ score of predictions was 0.415. Our checks on whether our outcomes of interest changed appreciably by altering outcome definition are summarized in Table 1. We found little evidence that they were affected by changes to inclusion of bouncebacks to ED. Figure 1 illustrates the stability of each of these outcomes over time. Compliance varied from a minimum monthly average of 63% in September 2023 to a peak of 94% in September 2021. Sensitivity also varied from a minimum of 28% in December 2021 to a maximum of 53% in April 2019. Relatively low PPV was observed across the period with an exception where it spiked to 71% in late 2022. There is, however, consistently high NPV observed over time. Specificity and accuracy were more stable over time, showing less variation in contrast to compliance or sensitivity. This stability is reflective of the high prevalence of patients who are not admitted to hospital. The relatively modest F_1_ score we estimated also reflects this and reflects poor sensitivity of nurse predictions. The exception to this pattern is that sensitivity was relatively high in the first month of prediction before it stabilized at a much lower baseline value in subsequent months.

**Figure 2:**
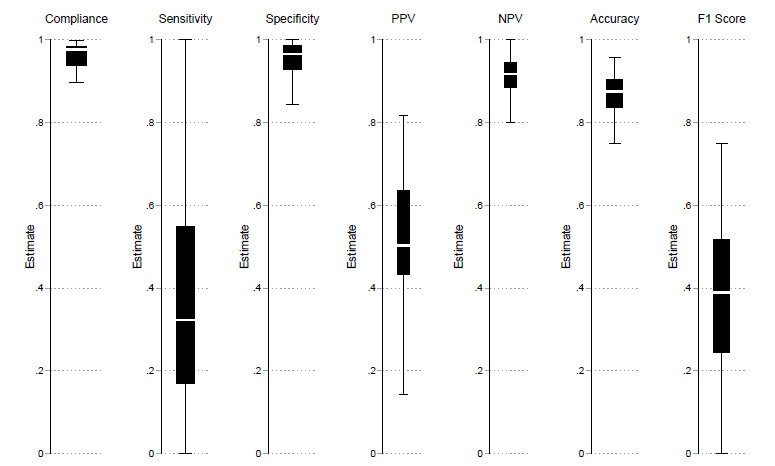
Variation in outcomes of interest across nurses. The center white line represents the median value; the edges of the box represent the 25th and 75th percentiles; and the whiskers indicate the upper and lower adjacent values. Estimates exclude values outside these adjacent values. *PPV*, positive predictive value; *NPV*, negative predictive value.

In line with our observations across time, compliance and specificity were similar across nurses (Figure 2). The lowest non-outlier compliance rates were ≈ 90%, indicating that non-compliance was concentrated in a minority of outlier nurses. Specificity also remained consistently high across most nurses in our sample with the lowest prediction specificity for a nurse being 84%. However, there was considerable variation in the sensitivities of nurse predictions, which ranged from 0 – 100%. This resulted in most nurses having prediction accuracies between 77 – 97%. Negative predictive values had a limited range between 82 – 100% whereas PPVs ranged between 18 – 91%. Nurse F_1_ scores ranged from 0 – 0.77.

**Figure 3:**
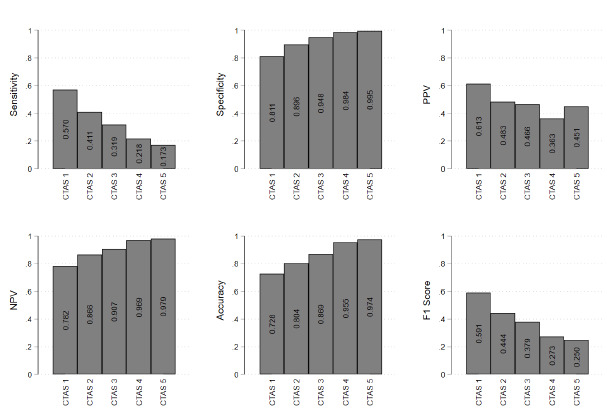
Outcomes of interest by triage scores. Canadian Triage and Acuity Scale scores range from 1–5: resuscitation (1), emergent (2), urgent (3), less urgent (4), and non-urgent visits (5). *CTAS*, Canadian Triage and Acuity Scale; *PPV*, positive predictive value; *NPV*, negative predictive value.

We found that predictive abilities also varied by patient type (Figure 3). We first examined nurses’ prediction accuracy by patient triage score. Nurses had a reduced compliance of 52% in making predictions for very sick patients, classified as CTAS 1, likely because some of these patients were paper triaged and predictions were not entered into the administrative data. For those patients who did not have resuscitation-level presentations, which includes CTAS 2 - 4, nurses predicted admission probability for ≈ 80% of all visits. Among those patients who received a prediction, we found a positive correlation between triage score severity and sensitivity, and a negative correlation between triage score severity and specificity. Higher triage severity and need for emergency resources means higher sensitivity and lower specificity. This resulted in a positive correlation between triage score and prediction accuracy and a negative correlation between triage score and F_1_ score.

We found that most conditions had high prediction specificity and corresponding low sensitivities (Supplemental Table 1). However, some conditions had comparatively high sensitivities as compared to other complaints. This included a cluster of complaints that related to altered levels of consciousness, confusion, bizarre behavior, and social- and patient-welfare concerns. Prediction accuracy was relatively high in a set of conditions that corresponded to low overall probability of hospital admission, such as bites and foreign bodies to the eye (Table 2). However, F_1_ scores were consistently poor with only the top 13 complaints demonstrating scores that could be considered average in terms of prediction. All remaining patient complaints had F_1_ scores that would be considered poor (Table 2).

## DISCUSSION

We estimated the sensitivity, specificity, PPV, NPV, accuracy, and the F_1_ score of triage nurses at an ED in the province of Ontario, Canada, to assess how effectively they predicted patient admission to hospital. Our contribution to the literature is twofold: 1) these estimates were not contaminated by Hawthorne effects, which are characteristic of previous prospective studies; and 2) we used a much larger data sample than previous studies. This allowed us to provide evidence on temporal, nurse, and patient heterogeneity in predictions.

We observed that this sample of nurses achieved reasonably high prediction accuracy for hospital admissions. Prediction accuracy was 85.8% and relatively stable over the entire period that we examined. However, the estimated F_1_-score was 0.415, which is poor and due to the relatively low sensitivity and PPV of predictions. Thus, high accuracy is predicated on a relatively high specificity among a group of patients that are more likely to be discharged from hospital. This high specificity is also possibly grounded in the relatively large numbers of non-emergent presentations (i.e., CTAS 5 - 3). Performance in predicting admissions to hospital was more modest, with sensitivities of 30–40%.

There are several explanations for this modest sensitivity. Triage nurses, as the point of first contact, have much less information to base predictions upon relative to other healthcare professionals in the ED. Prediction accuracy might improve if made by bedside nurses, who are able to use initial investigations and conduct a more involved physical exam. Another possibility is unfamiliarity with predictions. However, our accuracy results were stable over time, suggesting that nurses did not learn to improve their predictions with increased prediction practice. Feedback, training, and stakes may also be important to improve prediction sensitivity, but were absent in our setting. On this last point, predictions had no immediate impact on care within the ED and were largely supposed to improve inpatient care. Similarly, incentivization, also absent in our setting, has been demonstrated to improve performance in similar tasks. 22 However, poor sensitivity may be more of a general issue in ED care than one specific to triage nurses. Even highly trained physicians only predict patient outcomes with equivocal, or only slightly greater ability. 2

Our results, demonstrating that nurses had low sensitivity/high specificity and high NPV/low PPV, have implications for ED operations. High specificity and low PPV suggest the potential for over-triage, where nurses predict admission for patients who do not require admission. Where there is relatively low prevalence in need for admission, as in our setting, emergency physicians cannot necessarily trust a positive admission prediction from the nurse. Low sensitivity and high NPV suggest a simultaneous but opposite issue. Nurses are under-triaging and suggesting discharge for patients who should actually be admitted. In ED settings where admission is relatively rare, this may be useful in that most of the people the nurse identifies as not needing admission are probably safe for discharge. However, our results suggest that it is not reliably safe to trust the triage nurse’s discharge decision either.

Despite this paradoxical issue of simultaneous over- and under-triage and a poor F_1_ score for overall need for admission, our exploration of prediction heterogeneity suggests that emergency physicians should pay attention to certain predictions. Triage nurses are accurate at predicting admission for presentations related to mental health concerns, altered levels of consciousness, confusion, bizarre behavior, and social- and patient- welfare concerns. These had relatively higher sensitivities and could be used to accelerate admission planning. The inter-nurse variation we observed in our outcomes also suggests that particular nurses may be able to provide more accurate information to a physician about a patient’s discharge disposition, and so predictions from these nurses should be paid particular attention. Both these findings are novel and suggest nuance in understanding when to trust nursing admission predictions. It may be reasonable to delegate admission decisions for certain complaints and certain nurses under narrow circumstances.

Our findings are consistent with prior literature that indicates nurses are not able to predict patient admission with sufficient sensitivity, 10,14,23–26 and from an operations perspective suggests against the direct streamlining of patients to admission based on triage nurse predictions. This is contrary to some literature that suggests triage nurses may be able to achieve satisfactory levels of sensitivity to implement triage prediction programs. 11–13,15,27–29 It is unclear what drives this difference between our results and those found in the literature, but there are likely multiple factors. Site-specific circumstances and the nursing staff’s experience may have played a role. We also highlight that most of the prior literature is prospective, and nurses knew they were being monitored. Monitoring may alter prediction behavior and improve sensitivity and rule-in performance, possibly accounting for some of the superior sensitivities reported in earlier studies. 17 In support of this, we found some suggestive evidence of these Hawthorne effects. Sensitivity was much higher in our first month of observation when nurses were being told to produce predictions and the system was novel to them than in subsequent months.

Although triage nurses were unable to accurately predict patient admissions at our site, they had high NPV. This finding is consistent with previous literature, which demonstrates that triage nurses are better at predicting discharge. 11–13,23,26,28,29 This suggests that triage nurses may be useful in identifying patients who are *likely* to be discharged quickly. Emergency clinicians can then take a second, more involved examination and admit those patients that triage nurses may have under-triaged. This is already done using ED “see-and-treat” areas where patients deemed to require lower levels of care are streamed. Such streaming could help to reduce congestion and improve workflow in acute-care sections where most patients have been deemed to require admission. A version of this concept was demonstrated by Derlet et al (1995) who reported the successful diversion of 18% of adult ambulatory visits over a five-year period. This led to reductions in ED waiting times, the number of patients who left without care, and complications resulting from delayed care. 27 This also reduced costs without any deaths within 72 hours of patients being triaged. 27

Finally, we found evidence that triage nurses predicted well at the extremes of the triage distribution, having higher sensitivities in patients with low triage scores and who were more likely to be admitted, and higher specificities in patients with high triage scores and who were more likely to be discharged. This is consistent with previous literature, which suggests that prediction accuracy increases at the extremes of case severity. 12,13,23,25,28 While high and low admission rates for resuscitation and non-urgent triage scores, respectively, may make predicting dispositions easier, these categories only account for 5–9% of total ED visits. 30,31 Of total visits, 45–60% are categorized as the middle category, or urgent, which are considerably less predictable, with admission rates of 28.2–49.4%. 20,30,31 Our results on prediction accuracy by patient complaint reinforces this: triage nurses are most likely to accurately predict disposition among patients with complaints that are less likely to require admission to hospital. This result suggests that triage nurses may be most effective at making predictions when uncertainty is minimized.

## LIMITATIONS

We note several limitations of this study. First, because this was a single-site study, findings may not be generalizable to other settings. Second, due to the retrospective design of this study, information such as prediction confidence ratings were not collected, which we note impacted the accuracy of predictions in previous literature. Third, we did not have data explaining the 20% rate of non-compliance observed. It is possible that nurses selectively made predictions for cases in which they felt more confident, artificially inflating our reported sensitivity and specificity. Fourth, admission prediction is not included in current triage training and, consequently, formal implementation may be required to acquire the most accurate measurements. Fifth, while using bounceback presentations with admission allows us to account for incorrect discharge by the physician, it may result in underestimation of specificity and overestimation of sensitivity if the subsequent admission is for a reason unrelated to the index presentation. Lastly, although a retrospective trial limits influence of the Hawthorne effect, the absence of consequences or incentives for incorrect or accurate predictions, respectively, may have reduced the intentionality of predictions made by triage nurses and, in turn, accuracy.

## CONCLUSION

We found generally high accuracy but low F_1_ scores when triage nurses made admission predictions about patients at our site of interest in the province of Ontario, Canada. High accuracy stems from high specificities with modest sensitivities. We found notable variation in nurse accuracy and variation based on patient characteristics. These results suggest that nursing staff predictions could be useful in expediting some resource allocation decisions and improving flow in EDs.

## Supplementary Information



## Figures and Tables

**Table 1:** Estimates of outcomes of interest by changing bounceback criteria.

Outcome	Sensitivity	Specificity	PPV	NPV	Accuracy	F_1_ Score
Admission with 30-day bouncebacks (primary measure)	0.3665	0.9368	0.4791	0.9031	0.8587	0.4153
Admission with 7-day bouncebacks	0.3755	0.9353	0.4618	0.9102	0.8632	0.4142
Admission with 1-day bouncebacks	0.3888	0.9337	0.4424	0.9186	0.8688	0.4139
Admission at index visit	0.3892	0.9336	0.4415	0.919	0.869	0.4137

*PPV*, positive predictive value; *NPV*, negative predictive value

**Table 2:** Top and bottom 20 patient presentations by prediction accuracy and F_1_ score.

Bottom 20 complaints by accuracy				Top 20 complaints by accuracy			
Rank	Complaint	Accuracy	N	Rank	Complaint	Accuracy	N
1	Confusion	0.5964	721	59	Facial trauma	0.9340	485
2	Altered LOC	0.6207	1181	60	Neck swelling/pain	0.9340	849
3	General weakness	0.6611	4642	61	Isolated chest trauma – blunt	0.9369	317
4	Hyperglycemia	0.6829	360	62	URTI complaints	0.9386	277
5	Hypoglycemia	0.6832	123	63	Minor complaints NOS	0.9386	880
6	Shortness of breath	0.6832	6475	64	Sensory loss/paresthesia	0.9437	231
7	Concern for welfare	0.6842	209	65	Epistaxis	0.9453	585
8	Social problem	0.6887	106	66	Cough/congestion	0.9460	3018
9	Bizarre behavior	0.6994	316	67	Eye trauma	0.9506	263
10	Substance withdrawal	0.7132	258	68	Visual disturbance	0.9544	592
11	Abdominal mass/distension	0.7258	434	69	Upper extremity pain	0.9620	2396
12	Direct referral for consultation	0.7317	1092	70	Upper extremity injury	0.9659	5779
13	Overdose ingestion	0.7408	1065	71	Allergic reaction	0.9701	1204
14	Extremity weakness/symptoms of CVA	0.7445	1139	72	Rash	0.9738	1296
15	Blood in stool/melena	0.7484	1077	73	Prescription/medication request	0.9763	760
16	Depression/suicidal/deliberate self-harm	0.7517	584	74	Recheck eye	0.9771	218
17	Vomiting blood	0.7556	266	75	Burn	0.9805	257
18	Seizure	0.7556	1320	76	Eye pain	0.9867	602
19	Palpitations/irregular heartbeat	0.7712	1914	77	Dental/gum problem	0.9868	836
20	Edema, generalized	0.7739	115	78	Laceration/puncture	0.9885	3315

Bottom 20 complaints by F_1_ score				Top 20 complaints by F1 score			
Rank	Complaint	F_1_	N	Rank	Complaint	F_1_	N

1	Allergic reaction	0.0526	1204	59	Palpitations/irregular heartbeat	0.3559	1914
2	Epistaxis	0.0588	585	60	Abnormal lab/imaging results	0.3616	2838
3	Neck trauma	0.0909	251	61	Syncope/pre-syncope	0.3788	2305
4	Anxiety/situational crisis	0.0938	691	62	Bizarre behavior	0.3791	316
5	Laceration/puncture	0.0952	3315	63	Extremity weakness/symptoms of CVA	0.4680	1139
6	Vaginal bleed	0.1000	610	64	Edema, generalized	0.4800	115
7	Rectal/perineal pain	0.1081	346	65	Hypoglycemia	0.4935	123
8	Facial trauma	0.1111	485	66	Blood in stool/melena	0.5028	1077
9	Groin/pain mass	0.1176	322	67	Lower extremity injury	0.5102	6270
10	Depression/suicidal/deliberate self-harm	0.1212	584	68	Shortness of breath	0.5155	6475
11	Flank pain	0.1288	2517	69	Hyperglycemia	0.5379	360
12	Urinary retention	0.1429	641	70	General weakness	0.5527	4642
13	Other skin conditions	0.1481	294	71	Confusion	0.5813	721
14	Headache	0.1493	2426	72	Fever	0.5836	3151
15	Dental/gum problem	0.1538	836	73	URTI complaints	0.5854	277
16	Constipation	0.1777	591	74	Vomiting blood	0.6012	266
17	Medical device problem	0.1785	864	75	Social problem	0.6292	106
18	Prescription/medication request	0.1818	760	76	Direct referral for consultation	0.6387	1092
19	Visual disturbance	0.1818	592	77	Altered LOC	0.6601	1181
20	Hypertension	0.1835	827	78	Concern for welfare	0.6765	209

*LOC*, loss of consciousness; *CVA*, cerebrovascular accident, *URTI*, upper respiratory tract infection; *NOS, not otherwise specified*.
