# Supplementary material for: Accuracy of Triage Nurses in Predicting Patient Admissions: Retrospective, Large-sample Evidence from a Community Emergency Department
Source: West J Emerg Med. 2025 Sep 1;26(5):1179–87. doi: 10.5811/westjem.39947 (PMC12591623; doi:10.5811/westjem.39947)
Supplement: Supplementary file 1 [file wjem-26-1174-s001.docx]

| **Bottom 20 complaints by Specificity** | | | **Bottom 20 complaints by Sensitivity** | | | **Bottom 20 complaints by PPV** | | | **Bottom 20 complaints by NPV** | | |
| --- | --- | --- | --- | --- | --- | --- | --- | --- | --- | --- | --- |
| **Rank** | **Complaint** | **Specificity** | **Rank** | **Complaint** | **Sensitivity** | **Rank** | **Complaint** | **PPV** | **Rank** |  | **NPV** |
| 1 | Altered level of consciousness | 0.6221 | 1 | Allergic reaction | 0.0435 | 1 | Vaginal bleed | 0.0600 | 1 | Altered level of consciousness | 0.5292 |
| 2 | Confusion | 0.6746 | 2 | Epistaxis | 0.0435 | 2 | Anxiety/situational crisis | 0.0667 | 2 | Confusion | 0.5550 |
| 3 | Bizarre behaviour | 0.7385 | 3 | Laceration/puncture | 0.0800 | 3 | Allergic reaction | 0.0667 | 3 | General weakness | 0.6024 |
| 4 | Depression/suicidal/deliberate self-harm | 0.7566 | 4 | Neck trauma | 0.0833 | 4 | Depression/suicidal/deliberate self-harm | 0.0676 | 4 | Concern for patient's welfare | 0.6066 |
| 5 | General weakness | 0.7701 | 5 | Rectal/Perineal pain | 0.0833 | 5 | Epistaxis | 0.0909 | 5 | Social problem | 0.6818 |
| 6 | Hyperglycemia | 0.7767 | 6 | Facial trauma | 0.0870 | 6 | Neck trauma | 0.1000 | 6 | Hyperglycemia | 0.6846 |
| 7 | Overdose ingestion | 0.7771 | 7 | Groin pain/mass | 0.0909 | 7 | Laceration/puncture | 0.1176 | 7 | Shortness of breath | 0.6984 |
| 8 | Social problem | 0.7895 | 8 | Flank pain | 0.1071 | 8 | Facial trauma | 0.1538 | 8 | Hypoglycemia | 0.7222 |
| 9 | Direct referral for consultation | 0.8000 | 9 | Prescription/medication request | 0.1111 | 9 | Rectal/Perineal pain | 0.1538 | 9 | Direct referral for consultation | 0.7736 |
| 10 | Substance withdrawal | 0.8009 | 10 | Urinary retention | 0.1176 | 10 | Flank pain | 0.1615 | 10 | Vomiting blood | 0.7835 |
| 11 | Concern for patient's welfare | 0.8043 | 11 | Other skin conditions | 0.1176 | 11 | Groin pain/mass | 0.1667 | 11 | Abdominal mass/distention | 0.8023 |
| 12 | Extremity weakness/symptoms of CVA | 0.8210 | 12 | Headache | 0.1210 | 12 | Overdose ingestion | 0.1673 | 12 | Blood in stool/melena | 0.8082 |
| 13 | Hypoglycemia | 0.8228 | 13 | Dental/gum problem | 0.1250 | 13 | Hypertension | 0.1695 | 13 | Edema, generalized | 0.8191 |
| 14 | Shortness of breath | 0.8447 | 14 | Medical device problem | 0.1266 | 14 | Visual disturbance | 0.1765 | 14 | Substance withdrawal | 0.8358 |
| 15 | Seizure | 0.8473 | 15 | Constipation | 0.1455 | 15 | Urinary retention | 0.1818 | 15 | Fever | 0.8404 |
| 16 | Abdominal mass/distention | 0.8529 | 16 | Anxiety/situational crisis | 0.1579 | 16 | Headache | 0.1948 | 16 | Extremity weakness/symptoms of CVA | 0.8431 |
| 17 | Blood in stool/melena | 0.8577 | 17 | Upper extremity pain | 0.1622 | 17 | Substance misuse/intoxication | 0.2000 | 17 | Syncope/pre-syncope | 0.8461 |
| 18 | Palpitations/irregular heartbeat | 0.8636 | 18 | Minor complaints NOS | 0.1667 | 18 | Dental/gum problem | 0.2000 | 18 | Palpitations/irregular heartbeat | 0.8588 |
| 19 | Vomiting blood | 0.8686 | 19 | Isolated chest trauma - blunt | 0.1765 | 19 | Other skin conditions | 0.2000 | 19 | Abdominal pain | 0.8601 |
| 20 | Syncope/pre-syncope | 0.8822 | 20 | Neck swelling/pain | 0.1795 | 20 | Neck swelling/pain | 0.2258 | 20 | Seizure | 0.8607 |
| **Top 20 complaints by Specificity** | | | **Top 20 complaints by Sensitivity** | | | **Top 20 complaints by PPV** | | | **Top 20 complaints by NPV** | | |
| **Rank** | **Complaint** | **Specificity** | **Rank** | **Complaint** | **Sensitivity** | **Rank** | **Complaint** | **PPV** | **Rank** | **Complaint** | **NPV** |
| 59 | Headache | 0.9731 | 59 | Overdose ingestion | 0.4095 | 59 | Prescription/medication request | 0.5000 | 59 | Scrotal pain and/or swelling | 0.9609 |
| 60 | Visual disturbance | 0.9757 | 60 | Edema, generalized | 0.4138 | 60 | UTI complaints | 0.5000 | 60 | Multisystem trauma - blunt | 0.9609 |
| 61 | Facial trauma | 0.9762 | 61 | Lower extremity injury | 0.4276 | 61 | Recheck eye | 0.5000 | 61 | Eye trauma | 0.9611 |
| 62 | URTI complaints | 0.9764 | 62 | Shortness of breath | 0.4324 | 62 | Eye trauma | 0.5000 | 62 | Epistaxis | 0.9617 |
| 63 | Sensory loss/paresthesia | 0.9773 | 63 | Hypoglycemia | 0.4318 | 63 | Blood in stool/melena | 0.5403 | 63 | Sensory loss/paresthesia | 0.9641 |
| 64 | Cough/congestion | 0.9781 | 64 | Multisystem trauma - blunt | 0.4583 | 64 | Edema, generalized | 0.5714 | 64 | Cough/congestion | 0.9660 |
| 65 | Isolated chest trauma - blunt | 0.9800 | 65 | Blood in stool/melena | 0.4613 | 65 | Hypoglycemia | 0.5758 | 65 | Anxiety/situational crisis | 0.9721 |
| 66 | Epistaxis | 0.9822 | 66 | General weakness | 0.4732 | 66 | Hyperglycemia | 0.5966 | 66 | Upper extremity pain | 0.9737 |
| 67 | Minor complaints NOS | 0.9832 | 67 | Extremity weakness/symptoms of CVA | 0.4885 | 67 | Lower extremity injury | 0.6323 | 67 | Prescription/medication request | 0.9788 |
| 68 | Upper extremity injury | 0.9842 | 68 | Hyperglycemia | 0.4897 | 68 | Shortness of breath | 0.6408 | 68 | Visual disturbance | 0.9791 |
| 69 | Upper extremity pain | 0.9875 | 69 | Fever | 0.4951 | 69 | Confusion | 0.6506 | 69 | Upper extremity injury | 0.9811 |
| 70 | Eye trauma | 0.9880 | 70 | Bizarre behaviour | 0.5179 | 70 | Direct referral for consultation | 0.6574 | 70 | Recheck eye | 0.9815 |
| 71 | Allergic reaction | 0.9881 | 71 | URTI infections | 0.5217 | 71 | General weakness | 0.6650 | 71 | Allergic reaction | 0.9815 |
| 72 | Rash | 0.9913 | 72 | Confusion | 0.5274 | 72 | URTI complaints | 0.6667 | 72 | Rash | 0.9820 |
| 73 | Burn | 0.9921 | 73 | Vomiting blood | 0.5385 | 73 | Vomiting blood | 0.6806 | 73 | Depression/suicidal/deliberate self-harm | 0.9839 |
| 74 | Dental/gum problem | 0.9952 | 74 | Social problem | 0.5714 | 74 | Social problem | 0.7000 | 74 | Eye pain | 0.9867 |
| 75 | Recheck eye | 0.9953 | 75 | Depression/suicidal/deliberate self-harm | 0.5882 | 75 | Altered level of consciousness | 0.7062 | 75 | Vaginal bleed | 0.9875 |
| 76 | Laceration/puncture | 0.9954 | 76 | Concern for welfare | 0.5897 | 76 | Fever | 0.7105 | 76 | Burn | 0.9882 |
| 77 | Prescription/medication request | 0.9973 | 77 | Altered LOC | 0.6197 | 77 | Concern for patient's welfare | 0.7931 | 77 | Dental/gum problem | 0.9916 |
| 78 | Eye pain | 1.0000 | 78 | Direct referral for consultation | 0.6211 | 78 | Eye pain | 1.0000 | 78 | Laceration/puncture | 0.9930 |
